# Complement and coagulation cascades activation is the main pathophysiological pathway in early-onset severe preeclampsia revealed by maternal proteomics

**DOI:** 10.1038/s41598-021-82733-z

**Published:** 2021-02-04

**Authors:** Lina Youssef, Jezid Miranda, Miquel Blasco, Cristina Paules, Francesca Crovetto, Marta Palomo, Sergi Torramade-Moix, Héctor García-Calderó, Olga Tura-Ceide, Ana Paula Dantas, Virginia Hernandez-Gea, Pol Herrero, Nuria Canela, Josep Maria Campistol, Joan Carles Garcia-Pagan, Maribel Diaz-Ricart, Eduard Gratacos, Fatima Crispi

**Affiliations:** 1 BCNatal | Fetal Medicine Research Center (Hospital Clínic and Hospital Sant Joan de Déu), Institut d’Investigacions Biomèdiques August Pi i Sunyer (IDIBAPS), University of Barcelona, Barcelona, Spain; 2 Nephrology and Renal Transplantation Department, Hospital Clínic, Centro de Referencia en Enfermedad Glomerular Compleja del Sistema Nacional de Salud (CSUR), University of Barcelona, Barcelona, Spain; 3 Josep Carreras Leukaemia Research Institute, Hospital Clinic, University of Barcelona Campus, Barcelona, Spain; 4 Hematopathology, Centre Diagnòstic Biomèdic (CDB), Hospital Clinic, Institut d’Investigacions Biomèdiques August Pi i Sunyer (IDIBAPS), University of Barcelona, Barcelona, Spain; 5 Barcelona Endothelium Team (BET), Barcelona, Spain; 6 Barcelona Hepatic Hemodynamics Laboratory, Liver Unit, Hospital Clinic, Institut d’Investigacions Biomèdiques August Pi i Sunyer (IDIBAPS), University of Barcelona, Barcelona, Spain; 7 Centro de Investigación Biomédica en Red de Enfermedades Hepáticas y Digestivas (CIBEREHD), Health Care Provider of the European Reference Network on Rare Liver Disorders (ERN-Liver), Barcelona, Spain; 8 Department of Pulmonary Medicine, Hospital Clínic, Institut d’Investigacions Biomèdiques August Pi i Sunyer (IDIBAPS), University of Barcelona, Barcelona, Spain; 9 Biomedical Research Networking Center on Respiratory Diseases (CIBERES), Madrid, Spain; 10 Girona Biomedical Research Institute – IDIBGI, Girona, Spain; 11 Cardiovascular Institute, Hospital Clinic, Institut d’Investigacions Biomèdiques August Pi i Sunyer (IDIBAPS), University of Barcelona, Barcelona, Spain; 12 Eurecat, Centre Tecnològic de Catalunya, Centre for Omic Sciences (COS), Joint Unit Universitat Rovira i Virgili-EURECAT, Unique Scientific and Technical Infrastructures (ICTS), 43204 Reus, Spain; 13 Centre for Biomedical Research on Rare Diseases (CIBER-ER), Madrid, Spain; 14 Department of Maternal–Fetal Medicine (ICGON), Hospital Clínic, Sabino de Arana 1, 08028 Barcelona, Spain

**Keywords:** Pre-eclampsia, Proteomics, Protein-protein interaction networks, Molecular medicine

## Abstract

Preeclampsia is a pregnancy-specific multisystem disorder and a leading cause of maternal and perinatal morbidity and mortality. The exact pathogenesis of this multifactorial disease remains poorly defined. We applied proteomics analysis on maternal blood samples collected from 14 singleton pregnancies with early-onset severe preeclampsia and 6 uncomplicated pregnancies to investigate the pathophysiological pathways involved in this specific subgroup of preeclampsia. Maternal blood was drawn at diagnosis for cases and at matched gestational age for controls. LC–MS/MS proteomics analysis was conducted, and data were analyzed by multivariate and univariate statistical approaches with the identification of differential pathways by exploring the global human protein–protein interaction network. The unsupervised multivariate analysis (the principal component analysis) showed a clear difference between preeclamptic and uncomplicated pregnancies. The supervised multivariate analysis using orthogonal partial least square discriminant analysis resulted in a model with goodness of fit (R^2^X = 0.99, p < 0.001) and a strong predictive ability (Q^2^Y = 0.8, p < 0.001). By univariate analysis, we found 17 proteins statistically different after 5% FDR correction (q-value < 0.05). Pathway enrichment analysis revealed 5 significantly enriched pathways whereby the activation of the complement and coagulation cascades was on top (p = 3.17e−07). To validate these results, we assessed the deposits of C5b-9 complement complex and on endothelial cells that were exposed to activated plasma from an independent set of 4 cases of early-onset severe preeclampsia and 4 uncomplicated pregnancies. C5b-9 and Von Willbrand factor deposits were significantly higher in early-onset severe preeclampsia. Future studies are warranted to investigate potential therapeutic targets for early-onset severe preeclampsia within the complement and coagulation pathway.

## Introduction

Preeclampsia is a pregnancy-specific multisystem disorder, defined as new-onset elevated blood pressure accompanied by proteinuria after 20 weeks of gestation. It complicates 2–5% of pregnancies^1^ and is a leading cause of maternal and perinatal morbidity and mortality^2,3^. The exact pathogenesis of preeclampsia remains poorly defined. Disturbed placental function has long been associated with preeclampsia^4,5^. However, many other factors could play a role in preeclampsia evolution like maternal cardiovascular maladaptation^6,7^, angiogenic factors imbalance^8^ and oxidative stress. This multifactorial nature of preeclampsia explains partially its variable clinical presentation, ranging from mild cases that could be missed to very severe cases with a high risk of maternal or fetal death. Early-onset preeclampsia is less common than its late counterpart, however it is the most severe form that accumulates the majority of maternal and perinatal complications^9^. Due to the lack in understanding the pathophysiology of preeclampsia, there is no available specific treatment for this disease. The only current “cure” for preeclampsia is the delivery, which could be an acceptable choice for late-onset preeclampsia near term. Whereas, in early-onset cases, it is still a real challenge to balance the risk of severe maternal complications when continuing the pregnancy against the fetal prematurity risk when the decision is pregnancy termination. Thus, there is a clinical necessity for a treatment based on understanding the pathophysiology of early-onset preeclampsia to delay its evolution to a severe disease and postpone the need for iatrogenic preterm delivery.

Investigating biological processes in the maternal blood could help in better understanding the pathophysiology and identifying therapeutic targets for early-onset severe preeclampsia. In fact, proteins are essential parts and participate in virtually every process in the human body^10^. Proteomics techniques represent a strategy towards high-throughput analysis of the global set of proteins^11^. Recent reports have described the physiological pathways of interest for normal pregnancy adaptations through the gestation^12,13^. Though, applying proteomics analysis has the potential to detect maladaptation pathways associated with preeclampsia. In the last decade, few studies were conducted to explore the proteomic fingerprint of preeclampsia in maternal blood near delivery^14–20^, however their results are difficult to interpret due to including heterogenous cases of mild and severe preeclampsia as well as early and late-onset forms of this disorder. We hypothesized that focusing on a well-defined subgroup of early-onset severe preeclampsia may detect better the pathophysiological pathways involved in this subgroup by applying maternal blood proteomics. Thus, the aim of this study was to identify proteomic patterns associated with potential pathways that play a role in the pathogenesis of early-onset severe preeclampsia.

## Results

### Baseline and perinatal characteristics of the study population

Baseline characteristics and perinatal outcomes of the study populations are shown in Table 1. The study groups were similar in terms of maternal baseline characteristics. None of the 3 preeclamptic pregnancies achieved by assisted reproductive technologies involved ovum donation. As expected, preeclamptic pregnancies presented altered feto-placental Doppler parameters with significantly higher levels of creatinine, urea and uric acid compared to controls. Mean gestational age at delivery was 32 weeks in preeclampsia group with high rate (up to 80%) of cesarean sections and 100% admissions to the neonatal intensive care unit presenting subsequently one case of neonatal mortality. Hemolysis, elevated liver enzymes and low platelets (HELLP) syndrome has been diagnosed in two cases of preeclamptic pregnancies.

**Table 1:** Maternal, fetal and perinatal characteristics of the study population.

	Controls N = 6	Preeclampsia N = 14	p value
**Maternal characteristics**			
Age (years)	36.5 ± 2.9	34.1 ± 3.8	0.28*
Caucasian ethnicity	5 (83.3)	6 (42.9)	0.10^ƒ^
Pre-gestational BMI (kg/m^2^)	22.2 ± 2.7	24.1 ± 4.1	0.38*
Nulliparity	5 (83.3)	9 (64.3)	0.39^ƒ^
Previous preeclampsia	0 (0)	1 (7.1)	0.50^ƒ^
Chronic hypertension	0 (0)	1 (7.1)	0.50^ƒ^
Assisted reproductive technologies	0 (0)	3 (21.4)	0.22^ƒ^
Smoking during pregnancy	1 (16.7)	1 (7.1)	0.52^ƒ^
**Fetal ultrasound assessment**			
Gestational age at assessment (weeks)	26.6 ± 3.7	30.5 ± 2.6	0.013*
Estimated fetal weight (g)	994 ± 496	1187 ± 328	0.30*
Estimated fetal weight centile	34 (29–66)	1 (0–3)	< 0.001^ω^
Uterine arteries mean PI (z score)	− 0.53 ± 1.77	2.60 ± 2.08	0.002*
Umbilical artery PI (z score)	− 0.40 ± 0.32	1.27 ± 1.71	0.07*
Middle cerebral artery PI (z score)	0.13 ± 1.09	− 1.16 ± 0.99	0.027*
Cerebroplacental ratio (z score)	− 0.06 ± 0.89	− 1.93 ± 1.6	0.027*
Ductus venosus PI (z score)	− 1 ± 0.79	− 0.34 ± 1.29	0.44*
**Maternal biochemical assessment**			
Gestational age at blood draw (weeks)	30 ± 1.8	31.3 ± 1.8	0.16*
Creatinine (mg/dl)	0.42 ± 0.07	0.69 ± 0.17	0.001*
Urea (mg/dl)	15.4 ± 4.2	40.4 ± 16.7	0.002*
Sodium (mEq/l)	138.3 ± 1.2	136.4 ± 2.1	0.048*
Potassium (mEq/l)	4.13 ± 0.21	4.61 ± 0.23	0.001*
AST (u/l)	17 (15–23)	25 (16–48)	0.16^ω^
ALT (u/l)	15 (9–21)	27 (14–65)	0.07^ω^
GGT (u/l)	10 (7–11)	19 (15–28)	0.013^ω^
Uric acid (mg/dl)	3.33 ± 0.43	6.09 ± 1.25	< 0.001*
Glucose (mg/dl)	75.5 ± 7.1	87.8 ± 19.7	0.13*
Triglycerides (mg/dl)	178.8 ± 67.7	260.6 ± 97.8	0.06*
Total cholesterol (mg/dl)	277.2 ± 50.1	241.9 ± 54	0.16*
Fibrinogen (g/l)	4.57 ± 0.98	3.44 ± 1.05	0.07*
Platelets (10^3^/l)	271.8 ± 70.6	209.1 ± 71.9	0.09*
**Perinatal outcomes**			
Gestational age at delivery (weeks)	40.3 ± 1.1	32 ± 1.9	< 0.001*
Cesarean section	1 (16.7)	11 (78.6)	0.018^ƒ^
Male sex	2 (33.3)	6 (42.9)	0.69^ƒ^
Birthweight (g)	3531 ± 410	1287 ± 267	< 0.001*
Birthweight centile	58 (41–80)	0 (0–1)	< 0.001^ω^
APGAR score 5 min < 7	0 (0)	1 (7.1)	0.50^ƒ^
Umbilical artery pH	7.20 ± 0.07	7.20 ± 0.09	0.97*

Data are presented as mean ± standard deviation, median (interquartile range) or n (%) as appropriate.

*BMI* body mass index, *PI* pulsatility index, *AST* aspartate aminotransferase, *ALT* alanine aminotransferase, *GGT* gamma glutamyl transferase.

P value was calculated by Student’s *t* (*), Mann Whitney U (^ω^) or Fisher exact (^ƒ^) tests as appropriate.

### Proteomics results

**Figure 1:**
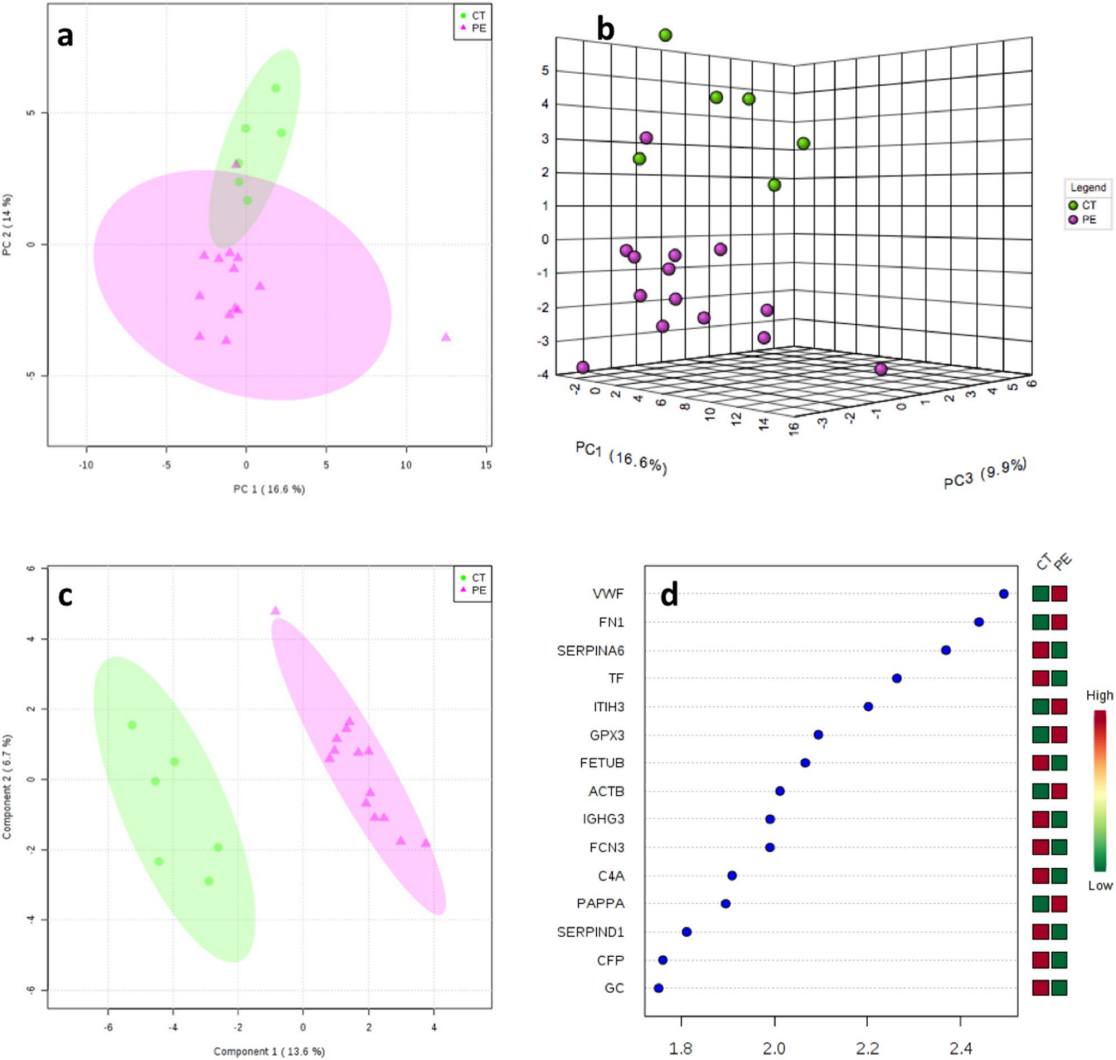
(**a**) 2-dimensional and (**b**) 3-dimensional principal component analysis (PCA) scores plots. (**c**) Partial least squares discriminant analysis (PLS-DA) scores plot between components 1 and 2. The explained variance is shown in brackets. Controls (CT) are presented in green circles and preeclampsia cases (PE) in violet triangles. (**d**) Top 15 most important proteins contributing to groups separation identified through PLS-DA ranked by variable importance in projection (VIP) scores. The right heatmap shows mean intensity variable in the respective group, with red and green indicating high and low protein levels, respectively. *VWF* Von Willebrand factor, *FN1* fibronectin, *SERPINA6* corticosteroid-binding globulin, *TF* serotransferrin, *ITIH3* inter-alpha-trypsin inhibitor heavy chain H3, *GPX3* glutathione peroxidase 3, *FETUB* FETUIN-B, *ACTB* actin, cytoplasmic 1, *IGHG3* immunoglobulin heavy constant gamma 3, *FCN3* ficolin-3, *C4A* complement C4-A, *PAPPA* pappalysin-1, *SERPIND1* heparin cofactor 2, *CFP* properdin, *GC* vitamin D-binding protein.

Out of 273 proteins identified, 158 were present in ≥ 70% of the samples (complete results dataset is provided in Supplementary Data). The principal component analysis (PCA) shown in Fig. 1a,b demonstrates the separation between preeclampsia and uncomplicated pregnancies. The first and second components explained 16.6% and 14% of the variance between cases and controls respectively (The main proteins involved in each component are provided in Supplementary Data). The partial least squares discriminant analysis (PLS-DA) analysis resulted in a clear separation between the groups (Fig. 1c). The top 15 most important proteins contributing to class separation are shown in Fig. 1d ranked by their contribution to distinguishing preeclampsia from controls. The greater the distance from the Y-axis, the greater is the contribution of a particular protein. The heatmap on the right side of this plot also indicates whether this protein’s concentration is increased or decreased in preeclampsia relative to controls. The model obtained by orthogonal projection to latent structures discriminant analysis (OPLS-DA) analysis showed a high goodness of fit (R^2^X = 0.995, p = 0.001) and a strong predictive ability (Q^2^Y = 0.797, *p* < 0.001, 1 predictive + 2 orthogonal components) i.e. the model explains more than 99% of the variation between the study groups with a predictive ability of 80%). HELLP cases did not cluster differently to other cases of preeclampsia.

**Figure 2:**
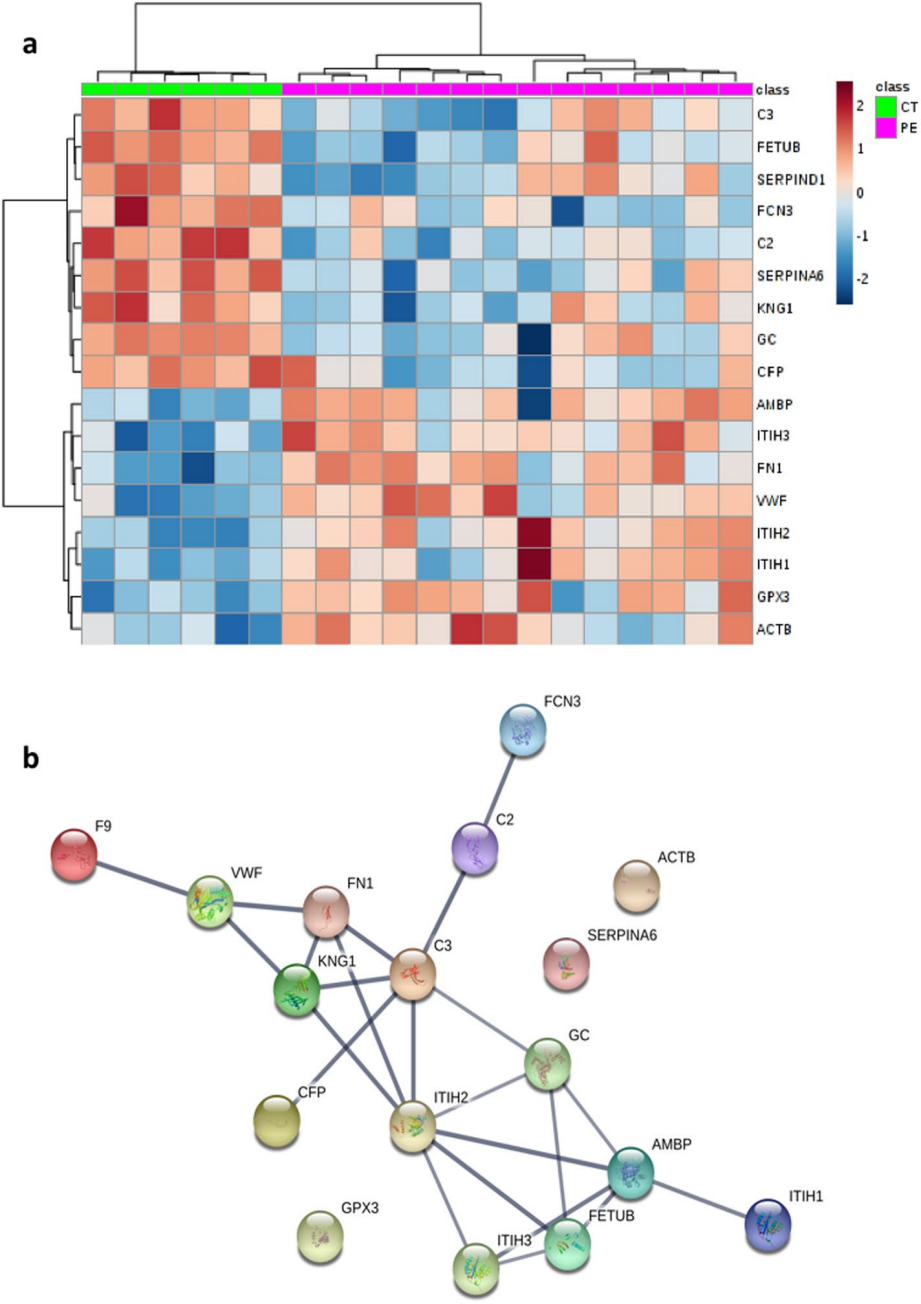
(**a**) Unsupervised hierarchical clustering based on the top 17 proteins selected from univariate analysis performed using Metaboanalyst 4.0 (http://www.metaboanalyst.ca/). A z-score transformation was performed on the intensity of each protein across all samples and each sample z-score is displayed in the heatmap. Proteins (in rows) and samples (in columns) are clustered by Euclidean distance and Ward linkage. (**b**) Protein–Protein Interaction network for these 17 proteins obtained using STRING database 11.00 (https://string-db.org/). Nodes represent proteins and edges interaction between proteins. The thickness of the edge indicates the degree of confidence prediction of the interaction. Only interactions with a high confidence score (> 0.7) were considered. *F9* coagulation factor IX, *C3* complement C3, *CFP* properdin, *VWF* Von Willbrand factor, *KNG1* kininogen-1, *FETUB* fetuin-B, *AMBP* protein AMBP, *ITIH1* inter-alpha-trypsin inhibitor heavy chain H1, *C2* complement C2, *SERPINA6* corticosteroid-binding globulin; *FN1* fibronectin type III domain containing, *ACTB* actin, cytoplasmic 1, *ITIH2* inter-alpha-trypsin inhibitor heavy chain H2, *GPX*3 Glutathione peroxidase 3, *GC* vitamin D-binding protein, *ITIH3* inter-alpha-trypsin inhibitor heavy chain H3, *FCN3* Ficolin-3.

**Figure 3:**
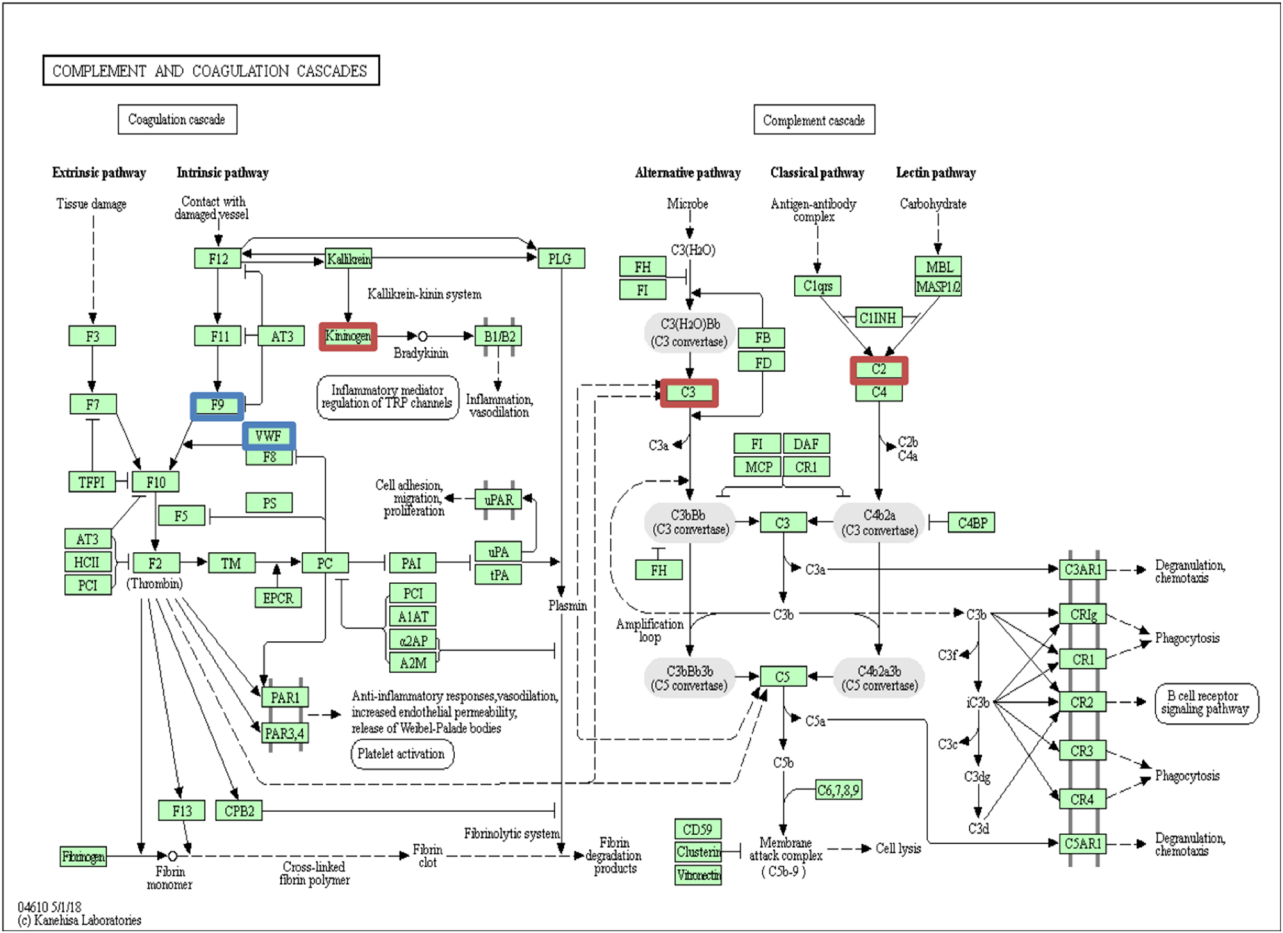
An illustration of the complement and coagulation cascades (homosapiens) from Kyoto Encyclopedia of Genes and Genome (KEGG) (https://www.genome.jp/kegg-bin/show_pathway?hsa04610). A total of 5 (out of 78) proteins were significantly enriched. Proteins that showed significantly lower and higher concentrations in preeclampsia are shown in red and blue respectively.

A total of 17 proteins were statistically different between the study groups by univariate analysis (Table 2), showing a good agreement with the multivariate results. Hierarchical clustering analysis (HCA) analysis considering these top 17 proteins showed 2 clusters corresponding to the study groups (Fig. 2a). The network generated using the 17 significant proteins is shown in Fig. 2b. Protein–protein interaction (PPI) enrichment p-value was < 1.62e−14 indicating that these proteins are not random. Pathway enrichment analysis revealed 5 significantly enriched pathways as shown in Table 3, the complement and coagulation cascades pathway was on top including 5 proteins (Fig. 3).

**Table 2:** Univariate analysis results, 17 proteins were significantly different in preeclampsia vs controls after false discovery rate correction (q-value < 0.05).

Uniprot	Protein name (gene name)	Controls Mean (SD)	Preeclampsia Mean (SD)	p-value	q-value	Preeclampsia/controls
P06681	Complement C2 (C2)	0.500 (0.245)	− 0.214 (0.247)	< 0.0001	0.0008	Down
Q9UGM5	Fetuin-B (FETUB)	0.649 (0.175)	− 0.278 (0.479)	< 0.0001	0.0008	Down
P02774	Vitamin D-binding protein (GC)	0.551 (0.128)	− 0.236 (0.492)	< 0.0001	0.0020	Down
P02751	Fibronectin type III domain containing (FN1)	− 0.766 (0.419)	0.328 (0.426)	< 0.0001	0.0020	Up
Q06033	Inter-alpha-trypsin inhibitor heavy chain H3 (ITIH3)	− 0.692 (0.463)	0.297 (0.399)	0.0001	0.0030	Up
P04275	Von Willebrand factor (VWF)	− 0.783 (0.467)	0.336 (0.483)	0.0001	0.0030	Up
P08185	Corticosteroid-binding globulin (SERPINA6)	0.744 (0.289)	− 0.319 (0.482)	< 0.0001	0.0030	Down
O75636	Ficolin-3 (FCN3)	0.626 (0.345)	− 0.268 (0.398)	0.0002	0.0030	Down
P19823	Inter-alpha-trypsin inhibitor heavy chain H2 (ITIH2)	− 0.550 (0.255)	0.236 (0.348)	0.0004	0.0063	Up
P22352	Glutathione peroxidase 3 (GPX3)	− 0.658 (0.327)	0.282 (0.491)	0.0005	0.0073	Up
P01042	Kininogen-1 (KNG1)	0.472 (0.306)	− 0.202 (0.385)	0.0013	0.0192	Down
P19827	Inter-alpha-trypsin inhibitor heavy chain H1 (ITIH1)	− 0.442 (0.193)	0.189 (0.393)	0.0016	0.0215	Up
P27918	Properdin (CFP)	0.553 (0.228)	− 0.237 (0.505)	0.0019	0.0231	Down
P01024	Complement C3 (C3)	0.391 (0.208)	− 0.168 (0.362)	0.0025	0.0286	Down
P02760	Protein AMBP (AMBP)	− 0.487 (0.273)	0.209 (0.519)	0.0046	0.0431	Up
P00740	Coagulation factor IX (F9) SERPIND1	− 0.336 (0.185)	0.144 (0.436)	0.0046	0.0431	Up
P60709	Actin, cytoplasmic 1 (ACTB)	− 0.632 (0.516)	0.271 (0.589)	0.0044	0.0431	Up

**Table 3:** Kyoto encyclopedia of genes and genome (KEGG) enrichment analysis of different proteins in preeclampsia vs controls.

Pathway	Count in gene set	Proteins involved		False discovery rate
		Increasing in preeclampsia	Decreasing in preeclampsia	
Complement and coagulation cascades	5 of 78	F9, VWF	KNG1, C3, C2	3.17e−07
Staphylococcus aureus infection	2 of 51		C3, C2	0.021
Pertussis	2 of 74		C3, C2	0.029
Systemic lupus erythematosus	2 of 94		C3, C2	0.034
Platelet activation	2 of 123	VWF, ACTB		0.045

*F9* coagulation factor IX, *C3* complement C3, *VW*F Von Willbrand factor, *KNG1* kininogen-1, *C2* complement C2, *ACTB* Actin, cytoplasmic 1.

**Figure 4:**
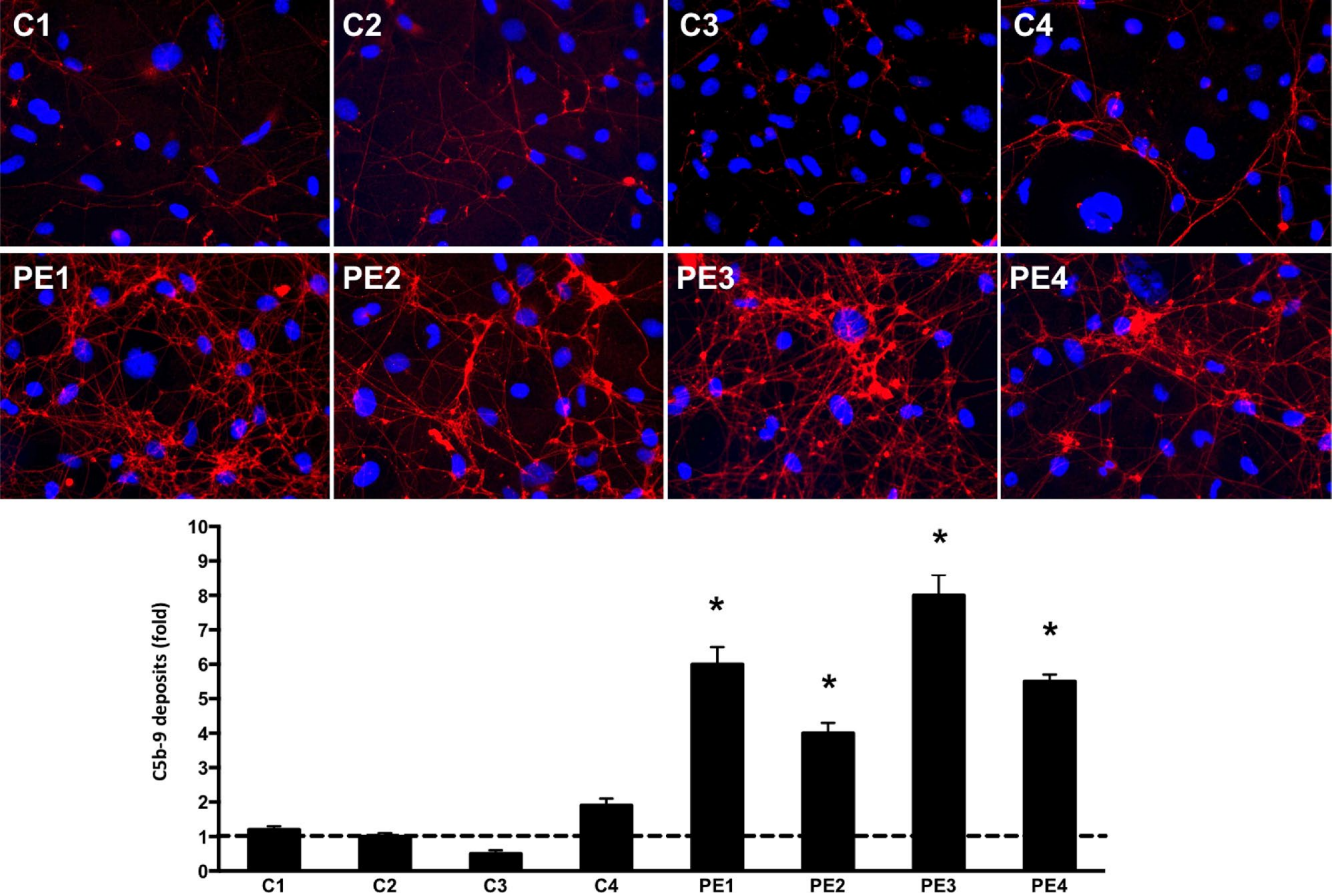
Representative microphotographs of the C5b-9 deposits (red) on endothelial cells in vitro (blue-labeled nuclei) induced by incubation with plasma from healthy pregnancies (controls, n = 4) and pregnancies complicated by preeclampsia (PE, n = 4). The bar chart represents the quantification of C5b-9 deposits. Whiskers represent the mean ± standard deviation. All the controls samples are within the normal range whereas the four pregnant women with PE show significant complement activation (*p < 0.05).

**Figure 5:**
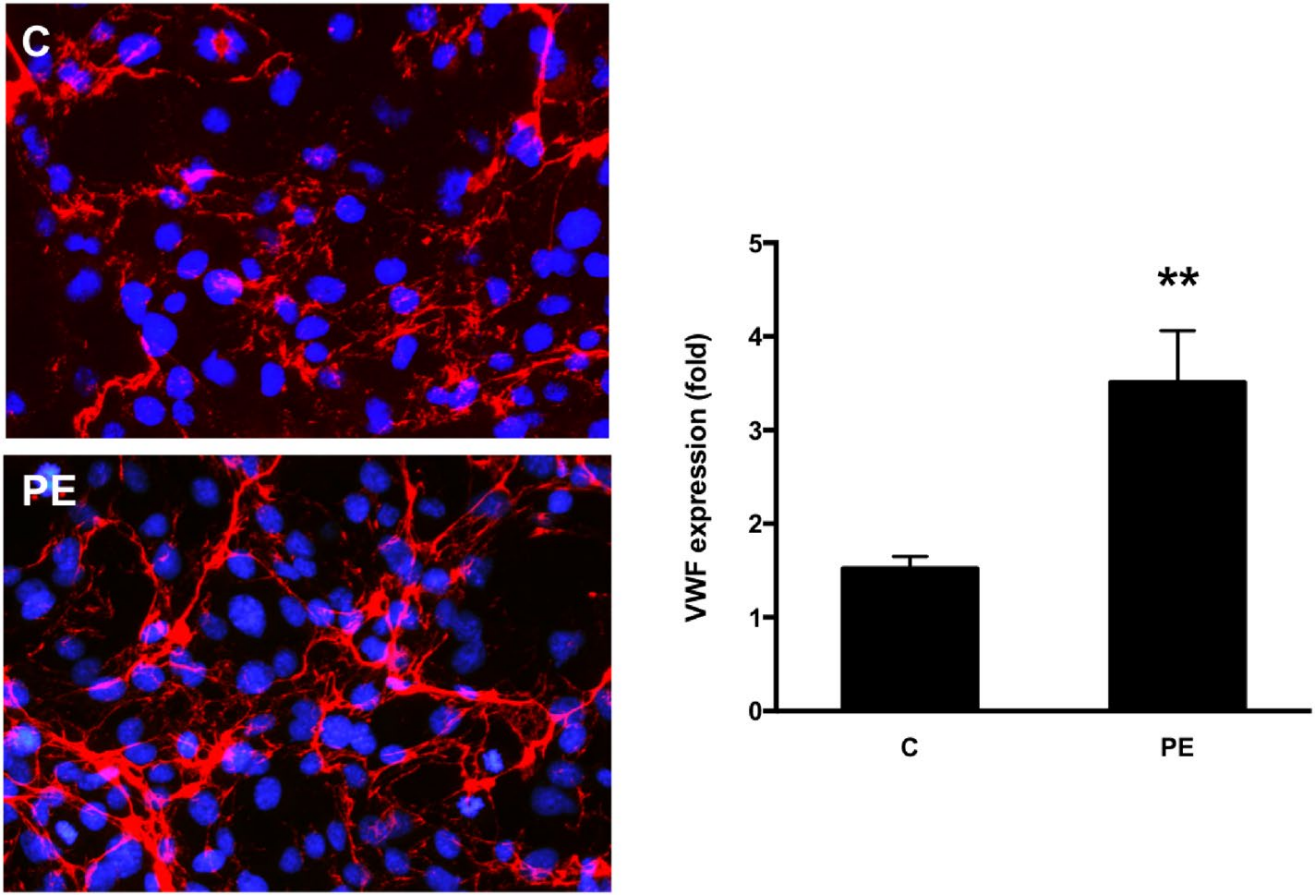
Representative microphotographs of the VWF deposits (red) on endothelial cells in vitro (blue-labeled nuclei) induced by incubation with plasma from healthy pregnancies and pregnancies complicated by preeclampsia. The bar chart represents the quantification of average VWF deposits. Whiskers represent the mean ± standard deviation. **p < 0.01 by Student’s t-test compared to controls.

The endothelial cells that were exposed to activated plasma from early-onset severe preeclampsia showed significantly higher deposits of C5b-9 complement complex and Von Willbrand factor (VWF) compared to controls, which reflects the activation of the complement and coagulation cascades in these patients and its effect on endothelial cells (Figs. 4, 5).

## Discussion

This study demonstrates the distinct maternal serum proteomic fingerprint in early-onset severe preeclampsia in comparison to uncomplicated pregnancies. This is the first study to show a wide set of circulating proteins and associated pathophysiological pathways which could provide potential therapeutic targets in this specific phenotype of preeclampsia.

The main differential pathway in early-onset severe preeclampsia was the complement and coagulation cascades activation. In addition, platelets activation was also significantly marked in this specific group of preeclampsia. Previous studies have demonstrated physiologic complement activation in normal pregnancy as a regulatory mechanism to facilitate the clearance of fetoplacental debris and protect against pathogens^21–24^. Further complement activation has been recognized in preeclampsia in the third trimester in line with heightened and more necrotic placental debris^25,26^. This excessive complement activation was correlated with the severity of preeclampsia and its association with fetal growth restriction^27,28^. Moreover, women with increased complement activation in early pregnancy were more likely to develop preeclampsia and adverse pregnancy outcome^29,30^. In accordance with our findings, a proteomic approach on pooled plasma from patients with preeclampsia and/or fetal growth restriction has showed that most proteins from the complement and coagulation cascades were identified in isolated fetal growth restriction and depleted in preeclampsia with and without fetal growth restriction^14^. Our group has also demonstrated that exposing endothelial cells in vitro to plasma from severe preeclampsia resulted in high deposition of the terminal complement pathway product known as membrane attack complex. This deposition was even stronger when the plasma was drawn from patients with HELLP syndrome and remained present at 6 weeks postpartum^31^.

The complement system is a conserved host defense system traditionally known for protecting against bacterial infection. Complement activation cascade leads to chemotaxis, immune-complex clearance and finally, the formation of membrane attack complex and cell lysis. Targeting excessive complement activation, particularly the terminal complement complex may be an effective strategy to prolong pregnancy in women with severe preeclampsia^32^. Of note, there are recent therapeutic options that block complement activation like eculizumab and ravulizumab which inhibit C5 and others blocking the cascade at different levels and thus preventing the formation of membrane attack complex^33,34^. Interestingly, eculizumab, which is already an approved treatment for both paroxysmal nocturnal hemoglobinuria and atypical hemolytic uremic syndrome, was utilized successfully as a temporizing treatment in a unique case of severe preeclampsia^34,35^. Our group has also demonstrated that blocking the membrane attack complex deposition on endothelial cells in vitro was achievable by adding eculizumab^31^.

In parallel to the complement pathway activation, the coagulation gets also initiated which in turn triggers platelet activity^36^. Activated platelets contribute to inflammation, immune responses and atherogenesis besides their central role in hemostasis^37–39^. They aggregate at the site of endothelial cell erosion, stimulating thrombus formation and promoting atherothrombotic disease^37^. Targeting these pathway by low molecular weight heparin or aspirin has failed as a treatment option once preeclampsia is diagnosed^1,40^.

Our results show that other pathophysiological pathways are also significantly enriched in early-onset severe PE, including staphylococcus aureus infection, pertussis and systematic lupus erythematosus which constitutes a known risk factor for preeclampsia^41^. These 3 pathways are all related to inflammatory processes and were enriched on the expenses of activated complement components C2 and C3. Preeclampsia has long been proposed as an excessive maternal inflammatory response to pregnancy^42^, since a systematic inflammatory profile similar to sepsis has been observed in preeclampsia^43^, which could be a cause or a consequence of generalized endothelial dysfunction^44^. Moreover, the most affected tissue in preeclampsia is maternal endothelium which is an integral part of the inflammatory network^45^. Additionally and in harmony with our observation of complement activation in preeclampsia, an accumulating evidence showed extensive cross-talk between inflammation and the complement system^46^ as well as the coagulation cascade, whereby coagulation affects considerably the inflammatory activity and vice versa^47^.

Our analysis demonstrated additionally other significantly different proteins in early-onset severe preeclampsia compared to controls. Fetuin-B is a protease inhibitor and a member of the fetuin family implicated in diverse functions, including osteogenesis and bone resorption, regulation of the insulin and hepatocyte growth factor receptors, and response to systemic inflammation^48^. Vitamin D-binding protein belongs to the albumin gene family and is able to bind the various forms of vitamin D^49^. Fibronectin type III domain is an evolutionary conserved protein domain that is widely found in animal proteins^50^. Inter-alpha-trypsin inhibitor heavy chains are main components together with AMBP protein of inter-alpha-trypsin inhibitors which function as protease inhibitors and have a role in inflammatory response^51^. Corticosteroid-binding globulin is a member of the serine protease inhibitor family and is implicated in steroid transport and delivery^52^. Ficolin-3 consists of a collagen-like domain and a fibrinogen-like domain and has lectin activity thereby it can activate the complement pathway aiding in host defense through the activation of the lectin pathway^53^. Glutathione peroxidase 3 functions in the detoxification of hydrogen peroxide, its lower activity both in the placenta and in maternal blood has been associated with severe PE^54,55^. Properdin is the only known positive regulator of complement activation that stabilizes the alternative pathway convertases^56^. Overall, most of these proteins are connected to the complement activation directly or indirectly through protease inhibition. It is well known that plasma protease inhibitors control a wide variety of physiological functions including blood coagulation, complement activation and aspects of the inflammatory response^57^.

This study has some strengths and limitations that merit a comment. We included prospectively a well characterized homogenous group of early-onset severe preeclampsia associated with fetal growth restriction, with no history of diabetes, autoimmune, renal or coagulation disorders. Furthermore, maternal blood samples were collected directly after confirmed preeclampsia diagnosis and at matched gestational age in controls and were processed and stored meticulously. In addition, our proteomic results were validated by quantifying C5b-9 and VWF deposits on endothelial cells exposed to activated plasma from an independent set of patients. However, we acknowledge the relatively small sample size included in this study and the need for investigating the identified pathways in other preeclampsia phenotypes. Besides, angiogenic factors that have been demonstrated to play a key role in preeclampsia and endothelial dysfunction^58^ were not detected by our approach. This is partly because different detection methods were used, angiogenic factors are usually detected by antibody-based techniques^59^ which are specific to one single protein at a time and have lower detection limits than mass spectrometry based proteomics approaches.

## Perspectives

Preeclampsia is considered to be responsible for up to 20% of the 13 million preterm births each year with high neonatal and long-term morbidity and mortality^60^. Given the high morbidity and mortality associated with severe cases of preeclampsia, any therapeutic strategy that allowed to avoid maternal complications and prolong pregnancy would have a clear benefit for the health of mothers and fetuses. Likewise, the implementation of these strategies would also have a clear economic impact for the national health system by reducing unnecessary hospital costs from caesarean sections and prolonged hospitalization in intensive care units. Our approach has identified the pathophysiological pathways involved in early-onset severe preeclampsia that could provide a better understanding of underlying etiology and reveal potential therapeutic targets for this phenotype of preeclampsia. Further research is warranted to allow the development of effective therapeutics that target complement without compromising its role in host defense^32^.

## Methods

### Study population

This was a nested case–control study within the project “Targeting endothelial dysfunction in highly prevalent diseases—PIE15/00027”. We included prospectively singleton pregnancies with a diagnosis of early-onset severe preeclampsia associated with fetal growth restriction who attended the Departments of Maternal–Fetal Medicine at BCNatal (Barcelona, Spain) between July 2016 and December 2017. Preeclampsia was defined as high blood pressure (systolic blood pressure ≥ 140 mmHg and/or diastolic blood pressure ≥ 90 mmHg on two occasions, at least four hours apart) with proteinuria (≥ 300 mg/24 h or protein/creatinine ratio ≥ 0.3) developed after 20 weeks of gestation^61,62^. Early-onset cases refer to those that needed elective delivery before 34 weeks of gestation indicated for severe preeclampsia^9^, which was considered upon presenting one or more of the following severity criteria^62^: blood pressure ≥ 160 mmHg systolic or ≥ 110 mmHg diastolic on two occasions at least 4 h apart, thrombocytopenia (< 100,000/mm^3^), impaired liver function (elevated blood concentrations of liver enzymes to twice normal concentration and/or severe persistent right upper quadrant or epigastric pain unresponsive to medication and not accounted for by alternative diagnoses), progressive renal insufficiency (serum creatinine concentration > 1.1 mg/dl), pulmonary edema, new-onset cerebral or visual disturbances. Pregnancy termination was by labor induction or cesarean section upon obstetric indication. Fetal growth restriction was defined as estimated fetal weight and birthweight below the 10th centile associated with either abnormal cerebroplacental ratio (< 5th centile) or abnormal uterine arteries mean pulsatility index (> 95th centile), or birthweight below the 3rd centile. Uncomplicated pregnancies with normotensive mothers and an appropriate for gestational age fetus-defined as estimated fetal weight and birthweight above the 10th centile- were randomly selected from our general population to be included as controls and matched with cases by maternal age, ethnicity, pre-gestational body mass index and gestational age at maternal blood draw (± 2 weeks). Estimated fetal weight and birthweight centiles were calculated according to local standards^63^. In all pregnancies, gestational age was calculated based on the crown-rump length at first trimester ultrasound^64^. Pregnancies with chromosomal/structural anomalies or intrauterine infection were excluded. The history of pregestational diabetes, autoimmune, renal or coagulation disorders were also considered excluding criteria. The study was conducted in accordance with the principles of the Helsinki declaration. The study protocol has been approved by the local ethics committee (HCB/2015/0585) and participating patients provided their written informed consent.

### Data collection

The following data were recorded upon enrollment: maternal age, ethnicity, pregestational body mass index, chronic hypertension, parity, obstetric history, mode of conception and smoking status.

Additionally, estimated fetal weight and feto-placental Doppler assessment was achieved in all the study participants. Ultrasound studies were performed using a Siemens Sonoline Antares (Siemens Medical Systems, Malvern, PA, USA) or a Voluson 730 Expert (GE Medical Systems, Milwaukee, WI, USA) with 6–4-MHz linear curved-array probes. Estimated fetal weight was calculated using the Hadlock formula^65^ and centile based on local reference curves^63^. Fetoplacental Doppler examination followed standardized guidelines^66^ included the uterine arteries^67^, the umbilical artery^68^, the fetal middle cerebral artery^68^ and the ductus venosus^69^ with the calculation of the cerebroplacental ratio^70^. Maternal biochemical profile was also assessed at the time of maternal blood draw including the evaluation of renal (creatinine, urea, sodium, potassium) and liver (aspartate aminotransferase, alanine aminotransferase, gamma glutamyl transferase) function, uric acid, glucose, triglycerides, total cholesterol, fibrinogen and platelets count.

At the time of delivery, gestational age, birthweight, birthweight centile, Apgar scores, umbilical artery pH, admissions to the neonatal intensive care unit and perinatal mortality were recorded.

### Maternal blood sampling

Peripheral maternal blood was obtained by venipuncture within 24–48 h of preeclampsia diagnosis and at matched gestational age for controls. The samples were incubated for 30 min at room temperature to allow clotting and subsequently centrifuged at 1500×*g* for 10 min at 4 °C to separate the serum from clots. Thereafter, serum samples were transferred to acetonitrile treated tubes and immediately stored at − 80 °C until assayed.

### Proteomic analysis

Before proteomic analysis, the depletion of the seven most abundant serum proteins (Albumin, Immunoglobulin G, antitrypsin, Immunoglobulin A, transferrin, haptoglobin and fibrinogen) was performed using the Human-7 Multiple Affinity Removal Spin cartridge from Agilent Technologies, to increase the number of identified proteins. Afterwards, samples were processed for tandem mass tag (TMT) before acquisition on a nanoscale liquid chromatography coupled to tandem mass spectrometry (nano LC–MS/MS) analysis with LTQ-Orbitrap Velos Pro from Thermo Fisher. Protein identification/quantification was performed on Proteome Discoverer software version 1.4.0.288 (Thermo Fisher Scientific) by Multidimensional Protein Identification Technology. On initial proteomic analysis, readers were blinded to patient’s status. Detailed methodology is provided as Supplementary Information.

### Validation of the proteomic results

To evaluate the activation of the complement and coagulation cascades, we assessed the deposits of C5b-9 complement complex and VWF on endothelial cells in vitro utilizing samples from an independent set of 4 healthy pregnancies and 4 cases of early-onset severe preeclampsia. Serum and plasma samples were obtained by centrifugation of nonanticoagulated blood and citrated blood (3000×*g*, 15 min), respectively, within 6 h of extraction. All samples were aliquoted and stored at − 80 °C until they were used, avoiding freeze/thaw cycles.

To assess C5b-9 deposits, we obtained activated plasma by adding control sera to patient citrated plasma (1:1) using a modified technique than the one described by Noris et al.^71^. The human dermal microvascular endothelial cell line [HMEC-1] (American Type Culture Collection) was seeded on glass coverslips and used confluent^72^. HMEC-1 were washed with test medium (HBSS without calcium or magnesium, 0.5% BSA; Life Technologies) and activated or not with 10 mM ADP (Sigma-Aldrich) (10 min, 37 °C). Cells were then incubated (4 h) with activated plasma diluted with test medium (1:2). Control samples were obtained by mixing healthy plasma from donors with pooled sera from controls. Cultures were then washed and fixed. For C5b-9 immunostaining, cells were treated with 2% BSA (1 h) and incubated with a rabbit anti-human complement C5b-9 complex (Calbiochem).

To assess VWF deposits, HMEC-1 were exposed to MCDB131 (Gibco-BRL, Madrid, Spain) supplemented with 20% sera from the patients under study or from their respective controls. Confluent cells were then washed (PBS, Gibco), fixed (PFA 4%, Electron Microscopy Sciences, Hatfield, PA, USA), permeabilized (Triton X-100 0,025% Sigma-Aldrich, St Louis, MO, USA), blocked (1% BSA for 30 min) and incubated with a specific antibody against human VWF (Dako, Denmark).

Then, in both assessments (C5b-9 and VWF), incubation with an Alexa-594 conjugated rabbit secondary antibody (Molecular Probes, NY, USA) and 4′,6-Diamidine-2′-phenylindole dihydrochloride (DAPI, Sigma-Aldrich, St Louis, MO, USA) for nuclei staining was performed. Micrographs were captured by fluorescent microscopy (Leica DM4000B) through a video camera (Leica DFC310FX) and analyzed using Fiji (ImageJ, Bethesda, Rockville, MD, USA)^73^. A total of 20 photographs were randomly obtained from each preparation. The area covered by fluorescent C5b-9 or VWF labelling was calculated and expressed as the average fold increase. All samples were tested at least three times.

### Statistical analysis

Clinical data were analyzed with the statistical software STATA 14.2 (StataCorp. 2015. Stata Statistical Software: Release 14. College Station, TX: StataCorp LP). Categorical data are presented as percentages and continuous data as mean ± standard deviation or median (interquartile range) according to their distribution (which was assessed for normality using the Kolmogorov–Smirnov test). Statistical analysis for continuous variables included the use of student *t*-tests for normally distributed data or Mann Whitney U tests in the rest. Fisher exact test was used for categorical variables. All reported p-values are 2 sided. Differences were considered significant when p < 0.05.

For proteomics data, statistical approach was performed using Metaboanalyst 4.0 (http://www.metaboanalyst.ca/). Initially, a multivariate modelling was applied including the use of unsupervised methods such as PCA, and supervised methods like PLS-DA and OPLS-DA. A variable importance in projection (VIP) plot, which is a visual representation of the importance of the particular proteins in discriminating the groups of interest, is provided. Secondly, for each protein a univariate Student’s *t*-test was performed and Benjamini–Hochberg method was used to adjust p values for multiple testing with consideration of 5% false discovery rate. An additional unsupervised HCA was performed based on the univariate results. Last, differential pathways were identified by PPI networks using STRING database 11.00 (https://string-db.org/)^74^ and enrichment analysis using Kyoto Encyclopedia of Genes and Genome (KEGG) database^75^. Detailed statistical approach is provided as Supplementary Data.

For C5b-9 and VWF deposits, the percentages of area covered were calculated as mean ± standard deviation. Statistical analysis was performed with raw data using Student’s t test for paired samples by the statistical software SPSS (IBM Corp. Released 2016. IBM SPSS Statistics for Windows, Version 24.0. Armonk, NY: IBM Corp). Results were considered statistically significant when p < 0.05.

All methods were carried out in accordance with relevant guidelines and regulations.

## Supplementary Information

Supplementary Information 1.

Supplementary Information 2.

## Data Availability

The proteomics quantification data reported in this study are available as supplementary information.
