# Supplementary material for: Complement and coagulation cascades activation is the main pathophysiological pathway in early-onset severe preeclampsia revealed by maternal proteomics
Source: Sci Rep. 2021 Feb 4;11:3048. doi: 10.1038/s41598-021-82733-z (PMC7862439; doi:10.1038/s41598-021-82733-z)
Supplement: Supplementary file 2 — Supplementary Information 2. [file 41598_2021_82733_MOESM2_ESM.pdf]

Complement and coagulation cascades activation is the main pathophysiological pathway in early-onset severe preeclampsia revealed by maternal proteomics

Lina Youssef[1]; Jezid Miranda[1]; Miquel Blasco[2]; Cristina Paules[1]; Francesca Crovetto[1]; Marta Palomo[3,4,5]; Sergi Torramade-Moix[4]; Héctor García-Calderó[6,7]; Olga Tura-Ceide[8,9,10], Ana Paula Dantas[11]; Virginia Hernandez-Gea[6,7]; Pol Herrero[12]; Nuria Canela[12]; Josep Maria Campistol[2,13]; Joan Carles Garcia-Pagan[6,7]; Maribel Diaz-Ricart[4,5]; Eduard Gratacos[1,13]*; Fatima Crispi[1,13]

[1]BCNatal | Fetal Medicine Research Center (Hospital Clínic and Hospital Sant Joan de Déu), Institut d'Investigacions Biomèdiques August Pi i Sunyer (IDIBAPS), University of Barcelona, Barcelona, Spain.

[2]Nephrology and Renal Transplantation Department, Hospital Clínic, Centro de Referencia en Enfermedad Glomerular Compleja del Sistema Nacional de Salud (CSUR), University of Barcelona, Barcelona, Spain.

[3]Josep Carreras Leukaemia Research Institute, Hospital Clinic, University of Barcelona Campus, Barcelona, Spain.

[4]Hematopathology, Centre Diagnòstic Biomèdic (CDB), Hospital Clinic, Institut d'Investigacions Biomèdiques August Pi i Sunyer (IDIBAPS), University of Barcelona, Barcelona, Spain.

[5]Barcelona Endothelium Team (BET).

[6]The Barcelona Hepatic Hemodynamics Laboratory, Liver Unit, Hospital Clinic, Institut d'Investigacions Biomèdiques August Pi i Sunyer (IDIBAPS), University of Barcelona, Barcelona, Spain.

[7]Centro de Investigación Biomédica en Red de Enfermedades Hepáticas y Digestivas (CIBEREHD), Barcelona, Spain.

[8]Department of Pulmonary Medicine, Hospital Clínic, Institut d'Investigacions Biomèdiques August Pi i Sunyer (IDIBAPS), University of Barcelona, Barcelona, Spain.

[9]Biomedical Research Networking Center on Respiratory Diseases (CIBERES), Madrid, Spain.

[10]Girona Biomedical Research Institute – IDIBGI, Girona, Spain.

[11]Cardiovascular Institute, Hospital Clinic, Institut d'Investigacions Biomèdiques August Pi i Sunyer (IDIBAPS), University of Barcelona, Barcelona, Spain.

[12]Eurecat, Centre Tecnològic de Catalunya, Centre for Omic Sciences (COS), Joint Unit Universitat Rovira i Virgili-EURECAT, Unique Scientific and Technical Infrastructures (ICTS), Reus, Spain.

[13]Centre for Biomedical Research on Rare Diseases (CIBER-ER), Madrid, Spain.

**Corresponding author*: Eduard Gratacós, Department of Maternal–Fetal Medicine (ICGON), Hospital Clínic, Sabino de Arana 1, 08028, Barcelona, Spain (e-mail: gratacos@clinic.cat)

Supplementary Information

Methods

Proteomics analysis

Before proteomic analysis, the depletion of the seven most abundant serum proteins (Albumin, Immunoglobulin G, antitrypsin, Immunoglobulin A, transferrin, haptoglobin and fibrinogen) was performed to increase the number of identified proteins. 12 μl of each sample were passed twice through the Human-7 Multiple Affinity Removal Spin cartridge from Agilent Technologies and the flow through fractions were collected for proteomic analysis following manufacturer's protocol. 30 μg of total protein (quantified by Bradford's method) were reduced with 4mM 1.4-Dithiothreitol for 1h at 37°C and alquilated with 8 mM iodoacetamide for 30 min at 25ºC in the dark. Afterwards, samples were overnight digested (pH 8.0, 37ºC) with sequencing-grade trypsin (Promega) at enzyme:protein ratio of 1:50. Digestion was quenched by acidification with 1% (v/v) formic acid and peptides were desalted on Oasis HLB SPE column (Waters) before tandem mass tag (TMT) 10-plex labelling (Thermo Fisher) following manufacturer's instructions.

To normalize all samples along the different TMT-multiplexed batches, a pool containing all the samples was included in each TMT batch. TMT 10-plex batches were desalted on Oasis HLB SPE columns before the nanoscale liquid chromatography coupled to tandem mass spectrometry (nanoLC-MS/MS) analysis.

Labelled and multiplexed samples were on-line fractionated in a strong cation exchange (SCX) nano-column (Agilent) by gradient salt pulsed sequential elution using ammonium acetate (NH4AcO). Thus, 6 fractions were analyzed for each TMT-plex using 0, 12.5, 25, 50, 100 and 500 mM NH4AcO. Each SCX fraction eluted were desalted on a trap nano-column (100 μm I.D.; 2cm length; 5μm particle diameter, Thermo Fisher Scientific, San José, CA, USA) and separated onto a C-18 reversed phase (RP) nano-column (75μm I.D.; 15cm length;

3μm particle diameter, Nikkyo Technos Co. LTD, Japan) on an EASY-II nano LC from Thermo Fisher. The chromatographic separation was performed with a 90 min gradient using Milli-Q water (0.1% formic acid) and acetonitrile (0.1% formic acid) as mobile phase at a flow rate of 300 nL/min.

Mass spectrometry analyses were performed on an LTQ-Orbitrap Velos Pro from Thermo Fisher by an enhanced FT-resolution MS spectrum (R=30,000 FHMW) followed by a data dependent FT-MS/MS acquisition (R=15,000 FHMW, 40% HCD) from the most intense ten parent ions with a charge state rejection of one and dynamic exclusion of 0.5 min.

Protein identification/quantification

For protein quantification, the ratios between each TMT-label against the pool label were used and quantification results were normalized based on protein median.

Protein identification/quantification was performed on Proteome Discoverer software v.1.4.0.288 (Thermo Fisher) by Multidimensional Protein Identification Technology (MudPIT). For protein identification, all MS and MS/MS spectra were analyzed using Mascot search engine (v.2.5). Two missed cleavages were allowed and an error of 0.02 Da for FT-MS/MS fragmentation mass and 10.0 ppm for a FT-MS parent ion mass were allowed. TMT-10plex was set as quantification modification and oxidation of methionine and acetylation of N-termini were set as dynamic modifications, whereas carbamidomethylation of cysteine was set as static modifications. The false discovery rate and protein probabilities were calculated by Perclorator.

On initial proteomic analysis, readers were blinded to patient's status.

Statistical analysis

For proteomics data, statistical approach was performed using Metaboanalyst 4.0 (http://www.metaboanalyst.ca/). Only those proteins that were present in ≥ 70% of the samples in at least one group were considered. To estimate missing values in the included

proteins, we used a Bayesian principal component analysis approach. Then, a log base 2 transformation was applied, and data were mean-centered for univariate analyses and Pareto scaled for multivariate analyses. Initially, a multivariate modelling was performed including the use of unsupervised methods such as principal component analysis (PCA), and supervised methods like partial least squares discriminant analysis (PLS-DA) and an orthogonal projection to latent structures discriminant analysis (OPLS-DA). A variable importance in projection (VIP) plot, which is a visual representation of the importance of the particular proteins in discriminating the groups of interest, is provided. To assess the significance of class discrimination whether it could be due to chance, a permutation test was performed applying 2000 permutations. For the selected model, the goodness of fit (R2X) and the predictive performance (Q2Y), which relate to the explained and predicted variance respectively, were calculated. All these methods were applied with a Pareto scaling.

Secondly, for each protein a univariate Student's *t*-test was performed and Benjamini-Hochberg method was used to adjust p values for multiple testing with consideration of 5% false discovery rate. An additional unsupervised hierarchical clustering analysis (HCA) was performed based on the univariate results.

Last, differential pathways were identified using a method based on protein-protein interaction networks and enrichment analysis using STRING database 11.00 (https://string-db.org/). Significantly different proteins based on the univariate analysis were used for the pathway analyses. The pathway networks were identified by exploring the interactions of pathway-enriched genes with the global human protein-protein interaction (PPI) network from the Search Tool for the Retrieval of Interacting Genes/proteins (STRING) database 11.0 (https://string-db.org/). We constructed an extended network based on a high confidence score of 0.7. In addition, an enrichment analysis was performed to identify the corresponding pathways Kyoto Encyclopedia of Genes and Genome (KEGG) database.

Results

The first component explained 16.6% of the variance between cases and controls and consisted mainly of Immunoglobulin heavy constant mu (IGHM), Hemoglobin subunit beta (HBB), Immunoglobulin lambda constant 2 (IGLC2), CD5 antigen-like (CD5L), Immunoglobulin lambda-like polypeptide 5 (IGLL5) which are related to blood pressure regulation, lipid synthesis, apoptosis and immune response. The second component explained 14% of the variance and consisted primarily of Serotransferrin (TRFE), Haptoglobin (HP), Immunoglobulin heavy constant gamma 3 (IGHG3), Pregnancy-specific beta-1-glycoprotein 9 (PSG9), Glutathione peroxidase 3 (GPX3), Corticosteroid-binding globulin (CBG) which have mainly a role in protecting cells from oxidative damage. The third component explained an additional 9.9% of the variance and consisted of Apolipoprotein-a (APOA), Apolipoprotein C-III (APOC3), Apolipoprotein C-II (APOC2) which have a multifaceted role in atherosclerosis, triglyceride homeostasis and lipoproteins metabolism.

Supplementary Table S1. Univariate analysis results for each protein. p-value is calculated with t-test as a default. p-value with (W) is calculated by the Wilcoxon Mann Whitney test. q-value is calculated after 5% false discovery rate correction.

Protein (Uniprot)	Controls mean (SD)	Preeclampsia mean (SD)	p-value	q-value	Preeclampsia /controls
P06681	0.500 (0.245)	-0.214 (0.247)	< 0.0001	0.0008	Down
Q9UGM5	0.649 (0.175)	-0.278 (0.479)	< 0.0001	0.0008	Down
P02774	0.551 (0.128)	-0.236 (0.492)	< 0.0001	0.0020	Down
P02751	-0.766 (0.419)	0.328 (0.426)	< 0.0001	0.0020	Up
P08185	0.744 (0.289)	-0.319 (0.482)	< 0.0001	0.0030	Down
Q06033	-0.692 (0.463)	0.297 (0.399)	0.0001	0.0030	Up
P04275	-0.783 (0.467)	0.336 (0.483)	0.0001	0.0030	Up
O75636	0.626 (0.345)	-0.268 (0.398)	0.0002	0.0030	Down
P19823	-0.550 (0.255)	0.236 (0.348)	0.0004 (W)	0.0063	Up
P22352	-0.658 (0.327)	0.282 (0.491)	0.0005	0.0073	Up
P01042	0.472 (0.306)	-0.202 (0.385)	0.0013	0.0192	Down
P19827	-0.442 (0.193)	0.189 (0.393)	0.0016	0.0215	Up
P27918	0.553 (0.228)	-0.237 (0.505)	0.0019	0.0231	Down
P01024	0.391 (0.208)	-0.168 (0.362)	0.0025	0.0286	Down
P60709	-0.632 (0.516)	0.271 (0.589)	0.0044	0.0431	Up
P02760	-0.487 (0.273)	0.209 (0.519)	0.0046 (W)	0.0431	Up
P00740	-0.336 (0.185)	0.144 (0.436)	0.0046 (W)	0.0431	Up
P05546	0.570 (0.358)	-0.244 (0.621)	0.0081	0.0711	Down
P01019	0.470 (0.429)	-0.201 (0.491)	0.0096	0.0798	Down
P02765	0.502 (0.450)	-0.215 (0.545)	0.0112	0.0869	Down

P0C0L4	0.600 (0.813)	-0.257 (0.535)	0.0116	0.0869	Down
P02763	-0.514 (0.615)	0.220 (0.514)	0.0127	0.0914	Up
P01008	0.433 (0.587)	-0.186 (0.414)	0.0144	0.0988	Down
P18428	0.498 (0.805)	-0.214 (0.455)	0.0205	0.1352	Down
P05543	0.351 (0.186)	-0.150 (0.470)	0.0224	0.1414	Down
P01011	-0.381 (0.401)	0.163 (0.474)	0.0247	0.1500	Up
P08697	0.344 (0.340)	-0.148 (0.443)	0.0266	0.1537	Down
P22692	-0.338 (0.577)	0.145 (0.326)	0.0272	0.1537	Up
P02655	-0.439 (0.657)	0.188 (0.684)	0.0326 (W)	0.1660	Up
Q96PD5	-0.177 (0.181)	0.076 (0.519)	0.0326 (W)	0.1660	Up
P02787	0.711 (0.954)	-0.305 (0.875)	0.0326 (W)	0.1660	Down
Q15582	0.346 (0.424)	-0.148 (0.480)	0.0431	0.2129	Down
P06396	0.263 (0.188)	-0.113 (0.604)	0.0505	0.2369	Down
P02750	0.336 (0.487)	-0.144 (0.465)	0.0510	0.2369	Down
Q13790	0.350 (0.361)	-0.150 (0.548)	0.0566	0.2555	Down
P0C0L5	0.507 (0.361)	-0.217 (0.848)	0.0621	0.2726	Down
Q92954	-0.391 (0.589)	0.167 (0.579)	0.0650	0.2776	Up
P00747	0.244 (0.144)	-0.105 (0.577)	0.0695 (W)	0.2823	Down
P22792	-0.305 (0.460)	0.131 (0.407)	0.0757 (W)	0.2823	Up
Q00887	0.477 (0.311)	-0.204 (1.092)	0.0757 (W)	0.2823	Down
Q13219	-0.596 (1.030)	0.255 (0.789)	0.0757 (W)	0.2823	Up
P51884	-0.286 (0.345)	0.123 (0.506)	0.0761 (W)	0.2823	Up
Q96IY4	-0.242 (0.175)	0.104 (0.434)	0.0794	0.2823	Up
P02790	0.264 (0.266)	-0.113 (0.463)	0.0805	0.2823	Down
P09871	-0.218 (0.165)	0.093 (0.396)	0.0834	0.2823	Up

Q9BXR6	0.256 (0.225)	-0.110 (0.663)	0.0836	0.2823	Down
Q16610	0.354 (0.302)	-0.152 (0.643)	0.0853	0.2823	Down
P02743	-0.348 (0.440)	0.149 (0.600)	0.0858	0.2823	Up
P02656	-0.447 (0.410)	0.192 (0.813)	0.0876	0.2823	Up
P01860	0.626 (1.406)	-0.268 (0.836)	0.0913	0.2885	Down
P05160	0.296 (0.272)	-0.127 (0.581)	0.1091	0.3379	Down
P05154	0.303 (0.479)	-0.130 (0.568)	0.1205	0.3660	Down
Q03591	0.464 (0.426)	-0.199 (0.955)	0.1241	0.3699	Down
O43866	0.232 (0.499)	-0.100 (0.927)	0.1297 (W)	0.3775	Down
P27169	-0.322 (0.665)	0.138 (0.568)	0.1314	0.3775	Up
P00751	0.278 (0.642)	-0.119 (0.471)	0.1384	0.3893	Down
P10909	-0.247 (0.355)	0.106 (0.507)	0.1404	0.3893	Up
P10643	-0.254 (0.426)	0.109 (0.517)	0.1500	0.4087	Up
P43251	0.296 (0.406)	-0.127 (0.616)	0.1528 (W)	0.4093	Down
P07357	0.224 (0.448)	-0.096 (0.452)	0.1631	0.4232	Down
P15169	-0.256 (0.568)	0.110 (0.496)	0.1634	0.4232	Up
Q14624	0.194 (0.223)	-0.083 (0.456)	0.1783	0.4545	Down
Q9Y6R7	-0.332 (0.533)	0.142 (0.756)	0.1821	0.4568	Up
O75882	0.200 (0.202)	-0.086 (0.496)	0.1942	0.4770	Down
P08519	-0.447 (1.155)	0.191 (0.907)	0.1994	0.4770	Up
P20742	-0.486 (1.089)	0.208 (1.063)	0.1999	0.4770	Up
P04196	0.247 (0.524)	-0.106 (0.556)	0.2023	0.4770	Down
P00742	-0.039 (0.184)	0.017 (0.765)	0.2074 (W)	0.4778	Up
P02647	-0.195 (0.418)	0.084 (0.445)	0.2087	0.4778	Up
P08603	0.206 (0.268)	-0.088 (0.649)	0.2158 (W)	0.4872	Down

P01034	-0.223 (0.564)	0.096 (0.524)	0.2382	0.5301	Up
P0DOY2	0.412 (1.022)	-0.177 (0.992)	0.2437	0.5339	Down
P04003	0.332 (0.779)	-0.142 (0.831)	0.2497	0.5339	Down
Q96KN2	-0.230 (0.413)	0.098 (0.620)	0.2534	0.5339	Up
P04264	0.334 (1.178)	-0.143 (0.657)	0.2566	0.5339	Down
P04278	0.274 (0.637)	-0.117 (0.706)	0.2584	0.5339	Down
P00746	-0.174 (0.359)	0.074 (0.471)	0.2655	0.5339	Up
P80108	0.222 (0.514)	-0.095 (0.586)	0.2668	0.5339	Down
P02768	-0.356 (0.976)	0.153 (0.435)	0.2670	0.5339	Up
P14151	0.195 (0.587)	-0.084 (0.491)	0.2867	0.5662	Down
P07359	0.203 (0.721)	-0.087 (0.483)	0.3011	0.5873	Down
P05155	-0.112 (0.360)	0.048 (0.497)	0.3119 (W)	0.5937	Up
P02753	-0.101 (0.581)	0.043 (0.798)	0.3119 (W)	0.5937	Up
P17936	-0.123 (0.204)	0.053 (0.561)	0.3198	0.6015	Up
P03952	0.179 (0.682)	-0.077 (0.454)	0.3335	0.6200	Down
P55058	-0.317 (1.039)	0.136 (0.316)	0.3392	0.6231	Up
P55056	-0.277 (0.714)	0.119 (0.873)	0.3434	0.6236	Up
P02746	0.133 (0.483)	-0.057 (0.378)	0.3536	0.6350	Down
B9A064	0.280 (0.508)	-0.120 (1.002)	0.3697	0.6563	Down
P02748	0.151 (0.652)	-0.065 (0.403)	0.3748	0.6580	Down
P00450	-0.130 (0.328)	0.056 (0.484)	0.4067	0.7061	Up
P81605	-0.288 (1.410)	0.123 (0.853)	0.4278	0.7347	Up
P02775	-0.099 (0.172)	0.043 (0.609)	0.4348	0.7386	Up
P49747	0.126 (0.514)	-0.054 (0.462)	0.4490	0.7492	Down
P02776	-0.152 (0.286)	0.065 (0.662)	0.4542	0.7492	Up

P04114	0.170 (0.624)	-0.073 (0.665)	0.4559	0.7492	Down
P02649	-0.155 (0.738)	0.067 (0.553)	0.4655	0.7492	Up
P43652	-0.142 (0.448)	0.061 (0.604)	0.4720	0.7492	Up
P68871	0.240 (0.556)	-0.103 (1.077)	0.4733	0.7492	Down
P05090	-0.147 (0.442)	0.063 (0.635)	0.4742	0.7492	Up
P12259	-0.149 (0.796)	0.064 (0.515)	0.4802	0.7512	Up
P69905	0.188 (1.033)	-0.081 (0.692)	0.5010	0.7658	Down
P01023	-0.126 (0.528)	0.054 (0.546)	0.5031	0.7658	Up
P06727	0.125 (0.348)	-0.053 (0.592)	0.5041	0.7658	Down
P00736	-0.028 (0.319)	0.012 (0.496)	0.5092 (W)	0.7662	Up
P01871	0.226 (0.847)	-0.097 (1.047)	0.5146	0.7671	Down
P02652	-0.099 (0.629)	0.042 (0.349)	0.5234	0.7729	Up
Q14520	0.138 (0.400)	-0.059 (0.715)	0.5365	0.7849	Down
P02747	0.139 (0.445)	-0.059 (0.693)	0.5466 (W)	0.7871	Down
P02671	-0.139 (0.761)	0.059 (0.622)	0.5480	0.7871	Up
Q15848	-0.133 (0.783)	0.057 (0.613)	0.5666	0.8065	Up
P05156	-0.077 (0.454)	0.033 (0.361)	0.5720	0.8070	Up
P04180	-0.115 (0.317)	0.049 (0.670)	0.5775	0.8075	Up
Q9Y5Y7	-0.126 (0.826)	0.054 (0.668)	0.6132	0.8364	Up
P49908	-0.094 (0.591)	0.040 (0.509)	0.6137	0.8364	Up
P00738	0.310 (1.953)	-0.133 (0.910)	0.6140	0.8364	Down
P35858	-0.090 (0.344)	0.038 (0.588)	0.6274	0.8465	Up
P06276	-0.078 (0.268)	0.034 (0.543)	0.6408	0.8465	Up
P01031	0.061 (0.349)	-0.026 (0.401)	0.6503	0.8465	Down
P48740	-0.076 (0.471)	0.033 (0.488)	0.6513	0.8465	Up

Q9UK55	0.087 (0.326)	-0.037 (0.631)	0.6572	0.8465	Down
O95445	0.086 (0.574)	-0.037 (0.550)	0.6582	0.8465	Down
P02745	0.099 (0.369)	-0.042 (0.598)	0.6590 (W)	0.8465	Down
O14791	-0.091 (0.357)	0.039 (0.760)	0.6979	0.8727	Up
P19652	-0.070 (0.453)	0.030 (0.544)	0.6980	0.8727	Up
P08571	0.069 (0.615)	-0.029 (0.480)	0.7035	0.8727	Down
P11464	-0.189 (1.674)	0.081 (0.647)	0.7162	0.8727	Up
P02749	-0.153 (0.726)	0.066 (0.635)	0.7181 (W)	0.8727	Up
P36980	0.061 (0.973)	-0.026 (0.650)	0.7181 (W)	0.8727	Down
Q04756	-0.066 (0.432)	0.028 (0.642)	0.7181 (W)	0.8727	Up
P03951	0.062 (0.266)	-0.027 (0.579)	0.7253	0.8748	Down
P07996	0.078 (0.678)	-0.033 (0.724)	0.7518	0.8999	Down
P29622	0.059 (0.649)	-0.025 (0.309)	0.7734	0.9187	Down
P23142	-0.062 (0.562)	0.027 (0.663)	0.7794	0.9190	Up
P00734	0.038 (0.314)	-0.016 (0.444)	0.7894	0.9239	Down
P36955	-0.041 (0.346)	0.018 (0.496)	0.7970	0.9260	Up
P02654	-0.103 (0.265)	0.044 (0.649)	0.8045 (W)	0.9278	Up
P01834	0.068 (0.574)	-0.029 (0.982)	0.8247	0.9443	Down
Q9NZP8	-0.030 (0.399)	0.013 (0.489)	0.8514	0.9634	Up
P04004	-0.022 (0.173)	0.009 (0.561)	0.8537	0.9634	Up
P04217	0.082 (0.135)	-0.035 (0.497)	0.8689 (W)	0.9643	Down
Q08380	0.042 (0.695)	-0.018 (0.781)	0.8731	0.9643	Down
P11226	-0.032 (0.324)	0.014 (1.038)	0.8835	0.9643	Up
P20061	0.029 (0.528)	-0.012 (0.642)	0.8916	0.9643	Down
P02766	0.023 (0.545)	-0.010 (0.466)	0.8922	0.9643	Down

P25311	-0.027 (0.465)	0.011 (0.681)	0.9023	0.9643	Up
P13671	0.050 (0.315)	-0.021 (0.548)	0.9044 (W)	0.9643	Down
P05452	0.013 (0.412)	-0.005 (0.486)	0.9044 (W)	0.9643	Down
P26927	-0.021 (0.602)	0.009 (0.546)	0.9158	0.9643	Up
P02741	0.037 (1.263)	-0.016 (1.058)	0.9241	0.9643	Down
P61626	0.020 (0.605)	-0.009 (0.628)	0.9244	0.9643	Down
P07225	0.019 (0.360)	-0.008 (0.655)	0.9276	0.9643	Down
P07358	0.012 (0.320)	-0.005 (0.529)	0.9417	0.9725	Down
P00748	0.150 (0.182)	-0.064 (0.741)	0.9680 (W)	0.9913	Down
P07360	0.005 (0.452)	-0.002 (0.397)	0.9725	0.9913	Down
P16070	0.004 (0.432)	-0.002 (0.666)	0.9842	0.9963	Down
P04070	-0.002 (0.254)	0.001 (0.622)	0.9921	0.9963	Up
P35542	-0.001 (0.732)	0.000 (0.564)	0.9963	0.9963	Up

Supplementary Table S2. Other proteins that were identified in less than 70% of the samples and thus excluded from statistical analysis.

**Protein (Uniprot)**					
P00739	P13645	P41222	Q92820	P15144	Q9BPX3
Q13046	P35527	Q02985	Q9BWP8	P01591	Q9NPR2
P0DJI8	Q9UHG3	Q99969	O14786	P03950	P59665
P35908	P01780	Q6NT52	P61769	P09486	Q96QP1
P0DJI9	P22891	O00391	A0A0A0MRZ8	P09327	P31151
Q15238	Q9UNN8	P24592	Q9HDC9	P01344	P11021
Q06141	P33151	P62328	P54108	P07195	Q9C0I1
P13727	Q9UQ72	P06702	Q9NQZ6	Q5SYB0	P17174
Q6EMK4	P01876	P13796	P05109	Q9ULH1	Q9NQ79
Q6UXB8	P40197	P33908	Q12933	P0DML2	Q13201
Q15113	P32119	P04075	Q8WW22	Q92974	Q6Q759
Q8WU03	O76031	P98160	P39060	Q5VUG0	P21817
O00187	O00541	Q9NPH3	Q13093	Q9BYX2	O15061
Q13103	Q86VB7	P19320	Q13683	O95236	Q92626
P05121	Q9H2A2	O43299	Q8N3J5	P11908	Q99996
Q16627	P01619	Q86UD1	P22460	Q00532	
Q16853	Q8TC59	Q15485	Q9NPJ8	Q14207	
Q12805	P24043	Q8IZC4	P36575	Q8ND07	
P01880	O00533	P43403	P62714	Q96JI7	
P00709	P09172	O75822	P80188	Q9UPN9	
